# 3,4-Dimethyl-*N*-(2,4,5-trimeth­oxy­benzyl­idene)-1,2-isoxazol-5-amine

**DOI:** 10.1107/S1600536810026966

**Published:** 2010-07-14

**Authors:** Abdullah M. Asiri, Salman A. Khan, Kong Wai Tan, Seik Weng Ng

**Affiliations:** a Chemistry Department, Faculty of Science, King Abdul Aziz University, PO Box 80203, Jeddah 21589, Saudi Arabia; b Department of Chemistry, University of Malaya, 50603 Kuala Lumpur, Malaysia

## Abstract

In the title compound, C_15_H_18_N_2_O_4_, the aromatic rings on the azomethine double bond are *trans* to each other [C—C=N—C torsion angle = −178.29 (12)°] and they are approximately coplanar, the dihedral angle between them being 5.0 (1)°.

## Related literature

For the spectroscopic characterization of a related Schiff base, see: Asiri *et al.* (2010 ▶).
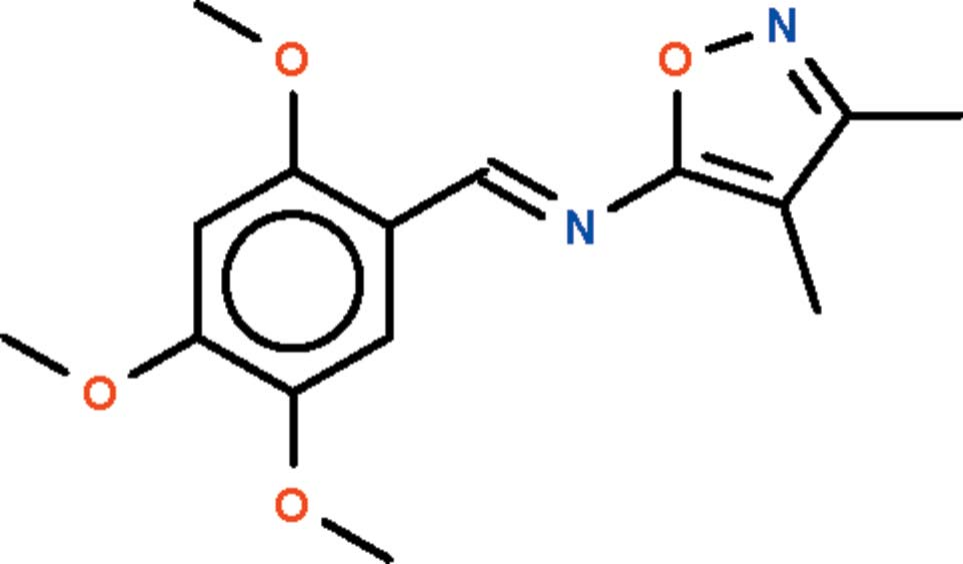


## Experimental

### 

#### Crystal data

C_15_H_18_N_2_O_4_
*M* *_r_* = 290.31
Triclinic, 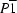

*a* = 6.6502 (5) Å
*b* = 10.9012 (8) Å
*c* = 11.2582 (8) Å
α = 63.463 (1)°
β = 83.078 (1)°
γ = 79.985 (1)°
*V* = 718.20 (9) Å^3^
*Z* = 2
Mo *K*α radiation
μ = 0.10 mm^−1^
*T* = 100 K
0.35 × 0.15 × 0.10 mm

#### Data collection

Bruker SMART APEX diffractometer
6732 measured reflections
3274 independent reflections
2660 reflections with *I* > 2σ(*I*)
*R*_int_ = 0.026

#### Refinement

*R*[*F* ^2^ > 2σ(*F* ^2^)] = 0.046
*wR*(*F* ^2^) = 0.139
*S* = 1.03
3274 reflections
195 parameters
H-atom parameters constrained
Δρ_max_ = 0.27 e Å^−3^
Δρ_min_ = −0.33 e Å^−3^

### 

Data collection: *APEX2* (Bruker, 2009 ▶); cell refinement: *SAINT* (Bruker, 2009 ▶); data reduction: *SAINT*; program(s) used to solve structure: *SHELXS97* (Sheldrick, 2008 ▶); program(s) used to refine structure: *SHELXL97* (Sheldrick, 2008 ▶); molecular graphics: *X-SEED* (Barbour, 2001 ▶); software used to prepare material for publication: *publCIF* (Westrip, 2010 ▶).

## Supplementary Material

Crystal structure: contains datablocks global, I. DOI: 10.1107/S1600536810026966/bt5294sup1.cif

Structure factors: contains datablocks I. DOI: 10.1107/S1600536810026966/bt5294Isup2.hkl

Additional supplementary materials:  crystallographic information; 3D view; checkCIF report

## Figures and Tables

**Fig. 1.**
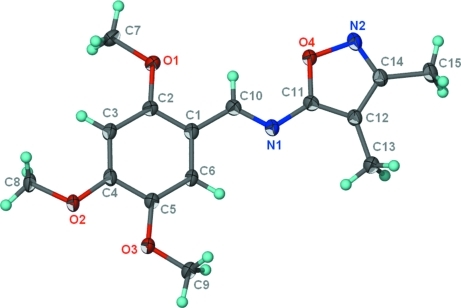
Anisotropic displacement ellipsoid plot (Barbour, 2001) of C15H18N2O4 at the 70% probability level; hydrogen atoms are drawn as spheres of arbitrary radius.

## supplementary crystallographic information

### Comment

The is yet no structural report on a Schiff-base condensation product involving 5-amino-3,4-dimethylisoxazole, a commerically available chemical. We recently reported the spectroscopic characterization of the *N*-ethylcarbazole-3-aldehyde condensation product of this amine (Asiri *et al.*, 2010). The 2,4,5-trimethyoxybenzaldehyde condensation product (Scheme I, Fig. 1) features an azomethine double-bond whose aromatic substituents are located in *trans* positions. The rings are coplanar [dihedral angle 5.0 (1)°].

### Experimental

5-Amino-3,4-dimethylisoxazole (0.36 g, 3.2 mol) and 2,4,5-trimethoxybenzaldehyde (0.62 g, 3.2 mol) were heated in methanol (15 ml) for 5 h. The solvent was removed and the solid material recrystallized from methanol to give the crystalline Schiff base.

### Refinement

Carbon-bound H-atoms were placed in calculated positions [C–H 0.95 to 0.98 Å, *U*(H) 1.2 to 1.5*U*_eq_(C)] and were included in the refinement in the riding model approximation.

### Crystal data

**Table:**

C_15_H_18_N_2_O_4_	*Z* = 2
*M**_r_* = 290.31	*F*(000) = 308
Triclinic, *P*1	*D*_x_ = 1.342 Mg m^−^^3^
Hall symbol: -P 1	Mo *K*α radiation, λ = 0.71073 Å
*a* = 6.6502 (5) Å	Cell parameters from 3842 reflections
*b* = 10.9012 (8) Å	θ = 3.1–28.3°
*c* = 11.2582 (8) Å	µ = 0.10 mm^−^^1^
α = 63.463 (1)°	*T* = 100 K
β = 83.078 (1)°	Prism, yellow
γ = 79.985 (1)°	0.35 × 0.15 × 0.10 mm
*V* = 718.20 (9) Å^3^	

### Data collection

**Table:**

Bruker SMART APEX diffractometer	2660 reflections with *I* > 2σ(*I*)
Radiation source: fine-focus sealed tube	*R*_int_ = 0.026
graphite	θ_max_ = 27.5°, θ_min_ = 2.0°
ω scans	*h* = −8→7
6732 measured reflections	*k* = −14→14
3274 independent reflections	*l* = −14→14

### Refinement

**Table:**

Refinement on *F*^2^	Primary atom site location: structure-invariant direct methods
Least-squares matrix: full	Secondary atom site location: difference Fourier map
*R*[*F*^2^ > 2σ(*F*^2^)] = 0.046	Hydrogen site location: inferred from neighbouring sites
*wR*(*F*^2^) = 0.139	H-atom parameters constrained
*S* = 1.03	*w* = 1/[σ^2^(*F*_o_^2^) + (0.0858*P*)^2^ + 0.1878*P*] where *P* = (*F*_o_^2^ + 2*F*_c_^2^)/3
3274 reflections	(Δ/σ)_max_ = 0.001
195 parameters	Δρ_max_ = 0.27 e Å^−^^3^
0 restraints	Δρ_min_ = −0.33 e Å^−^^3^

### Fractional atomic coordinates and isotropic or equivalent isotropic displacement parameters (Å^2^)

**Table:**

	*x*	*y*	*z*	*U*_iso_*/*U*_eq_	
O1	0.73187 (15)	0.64886 (10)	0.40960 (10)	0.0229 (2)	
O2	0.14017 (16)	0.97942 (10)	0.20733 (9)	0.0222 (2)	
O3	−0.06033 (15)	0.89581 (10)	0.43158 (9)	0.0225 (2)	
O4	0.72778 (15)	0.35850 (10)	0.86306 (9)	0.0212 (2)	
N1	0.42768 (18)	0.50963 (11)	0.76624 (11)	0.0190 (3)	
N2	0.79017 (19)	0.25984 (12)	0.99100 (11)	0.0223 (3)	
C1	0.4264 (2)	0.66108 (13)	0.53406 (13)	0.0184 (3)	
C2	0.5358 (2)	0.70964 (14)	0.41095 (13)	0.0185 (3)	
C3	0.4429 (2)	0.81575 (14)	0.29816 (13)	0.0191 (3)	
H3	0.5160	0.8470	0.2144	0.023*	
C4	0.2442 (2)	0.87494 (13)	0.30920 (13)	0.0184 (3)	
C5	0.1333 (2)	0.82831 (14)	0.43353 (13)	0.0187 (3)	
C6	0.2246 (2)	0.72188 (14)	0.54297 (13)	0.0188 (3)	
H6	0.1498	0.6889	0.6261	0.023*	
C7	0.8522 (2)	0.69800 (16)	0.28703 (14)	0.0248 (3)	
H7A	0.9905	0.6468	0.3013	0.037*	
H7B	0.8592	0.7968	0.2555	0.037*	
H7C	0.7891	0.6840	0.2205	0.037*	
C8	0.2345 (2)	1.02354 (15)	0.07629 (13)	0.0215 (3)	
H8A	0.1427	1.0982	0.0126	0.032*	
H8B	0.2623	0.9454	0.0530	0.032*	
H8C	0.3632	1.0572	0.0736	0.032*	
C9	−0.1756 (2)	0.85422 (16)	0.55545 (14)	0.0246 (3)	
H9A	−0.3094	0.9116	0.5426	0.037*	
H9B	−0.1021	0.8657	0.6195	0.037*	
H9C	−0.1944	0.7569	0.5894	0.037*	
C10	0.5224 (2)	0.55118 (14)	0.65057 (13)	0.0187 (3)	
H10	0.6573	0.5089	0.6419	0.022*	
C11	0.5270 (2)	0.40739 (13)	0.87469 (13)	0.0182 (3)	
C12	0.4552 (2)	0.34506 (13)	1.00327 (13)	0.0179 (3)	
C13	0.2444 (2)	0.36953 (15)	1.05885 (14)	0.0241 (3)	
H13A	0.1663	0.4497	0.9908	0.036*	
H13B	0.2527	0.3874	1.1360	0.036*	
H13C	0.1762	0.2876	1.0862	0.036*	
C14	0.6264 (2)	0.25434 (13)	1.07118 (13)	0.0187 (3)	
C15	0.6394 (2)	0.15920 (15)	1.21572 (14)	0.0242 (3)	
H15A	0.7754	0.1046	1.2323	0.036*	
H15B	0.5354	0.0969	1.2428	0.036*	
H15C	0.6161	0.2135	1.2670	0.036*	

### Atomic displacement parameters (Å^2^)

**Table:**

	*U*^11^	*U*^22^	*U*^33^	*U*^12^	*U*^13^	*U*^23^
O1	0.0179 (5)	0.0253 (5)	0.0175 (5)	0.0020 (4)	0.0022 (4)	−0.0046 (4)
O2	0.0224 (5)	0.0221 (5)	0.0123 (5)	0.0029 (4)	0.0006 (4)	−0.0011 (4)
O3	0.0194 (5)	0.0247 (5)	0.0143 (5)	0.0027 (4)	0.0013 (4)	−0.0029 (4)
O4	0.0193 (5)	0.0218 (5)	0.0152 (5)	0.0017 (4)	−0.0003 (4)	−0.0033 (4)
N1	0.0199 (6)	0.0176 (5)	0.0150 (5)	−0.0004 (4)	−0.0012 (4)	−0.0039 (4)
N2	0.0238 (6)	0.0207 (6)	0.0161 (6)	0.0018 (5)	−0.0034 (5)	−0.0036 (5)
C1	0.0197 (7)	0.0176 (6)	0.0150 (6)	−0.0017 (5)	−0.0004 (5)	−0.0051 (5)
C2	0.0168 (7)	0.0190 (6)	0.0175 (6)	−0.0003 (5)	0.0004 (5)	−0.0070 (5)
C3	0.0205 (7)	0.0186 (6)	0.0145 (6)	−0.0030 (5)	0.0016 (5)	−0.0045 (5)
C4	0.0217 (7)	0.0159 (6)	0.0133 (6)	−0.0009 (5)	−0.0010 (5)	−0.0030 (5)
C5	0.0184 (7)	0.0198 (6)	0.0153 (6)	−0.0015 (5)	0.0004 (5)	−0.0059 (5)
C6	0.0202 (7)	0.0194 (6)	0.0129 (6)	−0.0024 (5)	0.0013 (5)	−0.0043 (5)
C7	0.0191 (7)	0.0323 (8)	0.0198 (7)	−0.0035 (6)	0.0055 (5)	−0.0102 (6)
C8	0.0255 (7)	0.0218 (7)	0.0116 (6)	−0.0016 (5)	0.0013 (5)	−0.0034 (5)
C9	0.0218 (7)	0.0284 (7)	0.0165 (7)	−0.0006 (6)	0.0035 (5)	−0.0058 (6)
C10	0.0185 (7)	0.0173 (6)	0.0175 (6)	−0.0001 (5)	−0.0012 (5)	−0.0059 (5)
C11	0.0176 (7)	0.0168 (6)	0.0180 (6)	0.0006 (5)	−0.0016 (5)	−0.0065 (5)
C12	0.0196 (7)	0.0157 (6)	0.0160 (6)	−0.0004 (5)	−0.0015 (5)	−0.0053 (5)
C13	0.0210 (7)	0.0262 (7)	0.0182 (7)	0.0002 (5)	0.0012 (5)	−0.0054 (6)
C14	0.0223 (7)	0.0159 (6)	0.0162 (6)	−0.0004 (5)	−0.0020 (5)	−0.0059 (5)
C15	0.0285 (8)	0.0220 (7)	0.0163 (6)	0.0015 (6)	−0.0042 (6)	−0.0042 (5)

### Geometric parameters (Å, °)

**Table:**

O1—C2	1.3576 (16)	C7—H7A	0.9800
O1—C7	1.4330 (16)	C7—H7B	0.9800
O2—C4	1.3570 (16)	C7—H7C	0.9800
O2—C8	1.4316 (15)	C8—H8A	0.9800
O3—C5	1.3647 (17)	C8—H8B	0.9800
O3—C9	1.4269 (16)	C8—H8C	0.9800
O4—C11	1.3623 (16)	C9—H9A	0.9800
O4—N2	1.4171 (14)	C9—H9B	0.9800
N1—C10	1.2932 (18)	C9—H9C	0.9800
N1—C11	1.3755 (17)	C10—H10	0.9500
N2—C14	1.3197 (18)	C11—C12	1.3611 (18)
C1—C6	1.403 (2)	C12—C14	1.4148 (19)
C1—C2	1.4020 (18)	C12—C13	1.4951 (19)
C1—C10	1.4474 (18)	C13—H13A	0.9800
C2—C3	1.4001 (18)	C13—H13B	0.9800
C3—C4	1.3830 (19)	C13—H13C	0.9800
C3—H3	0.9500	C14—C15	1.4919 (18)
C4—C5	1.4171 (18)	C15—H15A	0.9800
C5—C6	1.3752 (18)	C15—H15B	0.9800
C6—H6	0.9500	C15—H15C	0.9800
			
C2—O1—C7	118.39 (11)	O2—C8—H8C	109.5
C4—O2—C8	117.96 (10)	H8A—C8—H8C	109.5
C5—O3—C9	116.69 (10)	H8B—C8—H8C	109.5
C11—O4—N2	107.97 (10)	O3—C9—H9A	109.5
C10—N1—C11	119.06 (12)	O3—C9—H9B	109.5
C14—N2—O4	105.38 (11)	H9A—C9—H9B	109.5
C6—C1—C2	119.14 (12)	O3—C9—H9C	109.5
C6—C1—C10	120.67 (12)	H9A—C9—H9C	109.5
C2—C1—C10	120.18 (13)	H9B—C9—H9C	109.5
O1—C2—C3	123.52 (12)	N1—C10—C1	121.13 (13)
O1—C2—C1	116.28 (12)	N1—C10—H10	119.4
C3—C2—C1	120.21 (12)	C1—C10—H10	119.4
C4—C3—C2	119.75 (12)	C12—C11—O4	110.29 (11)
C4—C3—H3	120.1	C12—C11—N1	128.95 (12)
C2—C3—H3	120.1	O4—C11—N1	120.70 (12)
O2—C4—C3	124.84 (12)	C11—C12—C14	103.98 (12)
O2—C4—C5	114.60 (12)	C11—C12—C13	127.53 (12)
C3—C4—C5	120.55 (12)	C14—C12—C13	128.48 (12)
O3—C5—C6	125.95 (12)	C12—C13—H13A	109.5
O3—C5—C4	114.91 (11)	C12—C13—H13B	109.5
C6—C5—C4	119.13 (13)	H13A—C13—H13B	109.5
C5—C6—C1	121.19 (13)	C12—C13—H13C	109.5
C5—C6—H6	119.4	H13A—C13—H13C	109.5
C1—C6—H6	119.4	H13B—C13—H13C	109.5
O1—C7—H7A	109.5	N2—C14—C12	112.38 (12)
O1—C7—H7B	109.5	N2—C14—C15	119.39 (13)
H7A—C7—H7B	109.5	C12—C14—C15	128.23 (13)
O1—C7—H7C	109.5	C14—C15—H15A	109.5
H7A—C7—H7C	109.5	C14—C15—H15B	109.5
H7B—C7—H7C	109.5	H15A—C15—H15B	109.5
O2—C8—H8A	109.5	C14—C15—H15C	109.5
O2—C8—H8B	109.5	H15A—C15—H15C	109.5
H8A—C8—H8B	109.5	H15B—C15—H15C	109.5
			
C11—O4—N2—C14	0.25 (14)	C4—C5—C6—C1	1.4 (2)
C7—O1—C2—C3	1.6 (2)	C2—C1—C6—C5	−0.3 (2)
C7—O1—C2—C1	−178.00 (12)	C10—C1—C6—C5	178.63 (12)
C6—C1—C2—O1	178.43 (12)	C11—N1—C10—C1	−178.29 (12)
C10—C1—C2—O1	−0.54 (19)	C6—C1—C10—N1	−2.4 (2)
C6—C1—C2—C3	−1.2 (2)	C2—C1—C10—N1	176.54 (13)
C10—C1—C2—C3	179.87 (12)	N2—O4—C11—C12	−0.41 (15)
O1—C2—C3—C4	−177.98 (12)	N2—O4—C11—N1	177.21 (11)
C1—C2—C3—C4	1.6 (2)	C10—N1—C11—C12	−177.34 (14)
C8—O2—C4—C3	6.2 (2)	C10—N1—C11—O4	5.53 (19)
C8—O2—C4—C5	−174.26 (12)	O4—C11—C12—C14	0.40 (15)
C2—C3—C4—O2	179.01 (12)	N1—C11—C12—C14	−176.98 (13)
C2—C3—C4—C5	−0.5 (2)	O4—C11—C12—C13	179.52 (13)
C9—O3—C5—C6	1.8 (2)	N1—C11—C12—C13	2.1 (2)
C9—O3—C5—C4	−178.54 (12)	O4—N2—C14—C12	0.00 (15)
O2—C4—C5—O3	−0.17 (18)	O4—N2—C14—C15	−179.83 (11)
C3—C4—C5—O3	179.42 (12)	C11—C12—C14—N2	−0.24 (16)
O2—C4—C5—C6	179.47 (12)	C13—C12—C14—N2	−179.36 (13)
C3—C4—C5—C6	−0.9 (2)	C11—C12—C14—C15	179.57 (14)
O3—C5—C6—C1	−179.04 (13)	C13—C12—C14—C15	0.5 (2)
